# Macromolecular Engineering of Poly(catechol) Cathodes towards High-Performance Aqueous Zinc-Polymer Batteries

**DOI:** 10.3390/polym13111673

**Published:** 2021-05-21

**Authors:** Nagaraj Patil, Jesus Palma, Rebeca Marcilla

**Affiliations:** Electrochemical Processes Unit, IMDEA Energy Institute, Avda. Ramón de la Sagra 3, 28935 Móstoles, Spain; jesus.palma@imdea.org

**Keywords:** redox polymers, organic electrode materials, zinc-metal batteries, aqueous batteries, polycatechol, high areal capacity, low temperature operativity

## Abstract

Aqueous zinc-polymer batteries (AZPBs) comprising abundant Zn metal anode and redox-active polymer (RAP) cathodes can be a promising solution for accomplishing viable, safe and sustainable energy storage systems. Though a limited number of RAPs have been successfully applied as organic cathodes in AZPBs, their macromolecular engineering towards improving electrochemical performance is rarely considered. In this study, we systematically compare performance of AZPB comprising Zn metal anode and either poly(catechol) homopolymer (named P(4VC)) or poly(catechol) copolymer (named P(4VC_86_-*stat*-SS_14_)) as polymer cathodes. Sulfonate anionic pendants in copolymer not only rendered lower activation energy and higher rate constant, but also conferred lower charge-transfer resistance, as well as facilitated Zn^2+^ mobility and less diffusion-controlled current responses compared to its homopolymer analogue. Consequently, the Zn||P(4VC_86_-*stat*-SS_14_) full-cell exhibits enhanced gravimetric (180 versus 120 mAh g^−1^ at 30 mg cm^−2^) and areal capacity (5.4 versus 3.6 mAh cm^−2^ at 30 mg cm^−2^) values, as well as superior rate capability both at room temperature (149 versus 105 mAh g^−1^ at 150 C) and at −35 °C (101 versus 35 mAh g^−1^ at 30 C) compared to Zn||P(4VC)_100_. This overall improved performance for Zn||P(4VC_86_-*stat*-SS_14_) is highly encouraging from the perspective applying macromolecular engineering strategies and paves the way for the design of advanced high-performance metal-organic batteries.

## 1. Introduction

Developing safe, cost-effective, viable, and high-performance rechargeable batteries is vital for realizing the sustainable energy-based low-carbon footprint society [1,2]. However, almost all of the commercial batteries, including the most efficient lithium-ion technologies contain toxic, scarce and/or environmentally unfriendly elements, which will hinder the desired target of achieving sustainable energy systems [3,4]. This triggers an ever-growing interest in finding alternative sustainable energy storage solutions. In this regard, aqueous batteries comprising organic electrode materials should provide ample opportunities owing to their inherent advantages in terms of abundance, harmlessness, safety, synthetic versatility, functional tunability, and low cost [5,6].

Among the reported aqueous batteries, rechargeable aqueous zinc-metal batteries (AZMBs) are one of the most promising candidates because of non-toxicity, greater abundancy, cost-effectiveness, and good compatibility of zinc anodes with water, in addition to their high specific capacity (820 mAh g^−1^) and high volumetric capacity (5851 mAh cm^−3^) [7,8,9,10,11,12,13,14,15,16,17,18]. In recent years, great progress has been made on building high-performance AZMBs mainly using inorganic intercalation/conversion cathode materials, such as metal oxides (manganese, vanadium, etc.) and Prussian blue analogues, and the current research trend continues mostly focusing on improving these systems. However, the combination of Zn anodes with more sustainable organic-based cathodes has been much less explored to date. Potentially, the organic cathodes can be considered as a sustainable alternative to conventional inorganic electrode materials owing to their natural abundance, less geopolitical constraints on resource availability, ease of synthesis, and can also be sometimes bio-based and biodegradable. 

Among the different categories of organic active-materials (small molecules, supramolecular assemblies, cross-linked networks, etc.), redox-active polymers (RAPs) are one of the most versatile organic electrode materials (OEMs) because of synthetic flexibility, composition tunability, controllability of topology, etc., to name just a few [19,20,21,22,23,24,25,26,27,28,29]. Though the techniques of integration of redox functionalities into a polymer backbone have become standard tools to impart an enhanced chemical, dimensional and mechanical stability to the redox units, the benefits of macromolecular engineering were scarcely exploited to its full potential and most of the RAPs were presented in their simplest form. However, in our previous works, we employed reversible addition–fragmentation chain transfer [30] and cobalt-mediated radical polymerization [31,32] approaches to design innovative ion-conducting RAPs, in the form of copolymers and homopolymers, in the former and latter examples, respectively, with controlled molar mass and tunable chemical structure and functionality. These RAPs demonstrated greatly improved Li-ion storage performance (superior capacity utilization at high currents and high mass loadings) compared to their poor ion-conducting RAPs counterparts owing to the enhanced ion mobility in the bulk of electrode. It is important to note that this lack of sufficient ion mobility is often a limiting factor to get practical high mass loading electrodes (of high areal capacity) with good performance at high currents. Lately, such synthetic strategies have been extended to design dual redox-active and ion conducting RAPs for organic electrochemical energy storage systems [33,34,35,36,37].

Although numerous RAPs have been successfully applied as OEMs in different rechargeable battery technologies, only a limited number of redox polymers were positively evaluated in AZMB (see Table S1) [38,39,40,41,42,43,44,45,46,47,48,49,50]. Here onwards we use an acronym AZPB for the AZMB comprising a polymer cathode and Zn metal anode. Most of these RAPs belong to *n*-type organics (mostly, quinones or imides) and rarely employed *p*-type aniline, tetrathiafulvalene and nitroxide radical-based polymers. Among them, quinone based polymers generally exhibited high specific capacities (as high as 372 mAh g^−1^), but suffered from low voltage output (below 1 V) and poor cycle life. In contrast, *p*-type RAPs delivered high voltage output (as high as 1.58 V) and good cycle performance but demonstrated low specific capacities (typically, <150 mAh g^−1^). In our most recent work, we pushed the limits of AZPBs to a new level for *n*-type organics by employing high performance poly(catechol) copolymer, P(4VC_86_-*stat*-SS_14_) cathode in a novel 4 m Zn(TFSI)_2_ aqueous electrolyte [51]. This battery simultaneously achieved high gravimetric capacity (324 mAh g^−1^), high voltage output (1.1 V), remarkable rate capability (98 mAh g^−1^ at 450 C) and extremely high cyclability with 83% capacity retention of over an extended 48,000 cycles.

**Figure 1:**
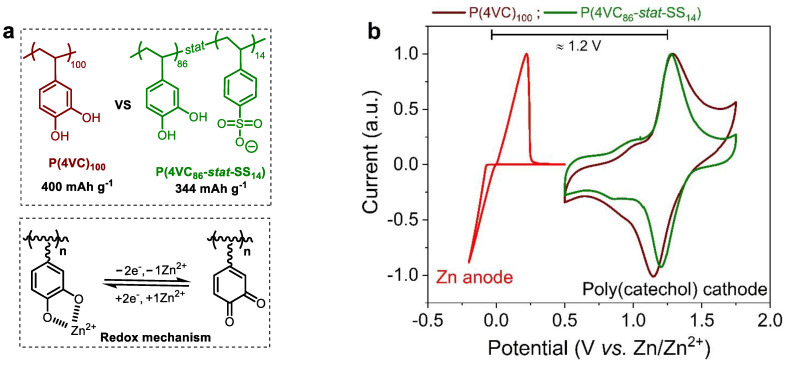
Design of Zn||poly(catechol) aqueous battery. (**a**) Chemical structure of poly(catechol) homo and copolymers, along with simplified redox mechanism; (**b**) CVs of Zn anode and P(4VC)_100_ or P(4VC_86_-*stat*-SS_14_) cathodes in 4 m Zn(TFSI)_2_-H_2_O aqueous electrolyte. The CVs were recorded in a three-electrode configuration with polymer-based electrode (or Ti foil), Zn wire and Zn foil as the working, reference and counter electrodes, respectively at 5 mV s^−1^.

In this article, we justify the selection of P(4VC_86_-*stat*-SS_14_) over its homopolymer analogue P(4VC)_100_ to construct high performance AZPBs (see Figure 1a for chemical structure of homo and copolymers). First, we study the comparative rate performance of Zn||P(4VC)_100_ versus Zn||P(4VC_86_-*stat*-SS_14_) full-cells at different temperatures, ranging from +25 to −35 °C. Second, cyclic voltammetry (CV) and electrochemical impedance spectroscopy (EIS) studies are employed to understand the electrochemical performance differences between these polymers. Interestingly, it is demonstrated that the enhanced rate performance for the copolymer over the homopolymer was linked to a combination of lower activation energy, higher redox reaction rate and improved Zn^2+^ ion mobility. Third, by exploiting these electrochemical enhancement features, we demonstrate the construction of practical organic electrodes employed in a Zn||P(4VC_86_-*stat*-SS_14_) battery with high areal capacity (5.4 mAh cm^−2^; one of the highest reported value till date for RAPs in AZPBs) with high rate capability (1.5 mAh cm^−2^ at 10 C) and good cycling stability (74% capacity retention over 400 cycles at 1 C).

## 2. Materials and Methods

Materials: Zinc bis(trifluoromethanesulfonyl)imide (Zn(TFSI)_2_, 99.5%, Solvionic, Toulouse, France), anhydrous 1-methyl-2-pyrrolidone (NMP, ≥99.5%, Sigma-Aldrich, Saint Louis, MO, USA), thin multi-walled carbon nanotubes (CNTs; Elicarb^®^ MW, Thomas-Swan, Consett, UK) were used as received. Zinc (Zn, 99.98%, Alfa Aeser, Haverhill, USA) foil was polished with sand paper to remove surface oxide layer, washed successively with 3% HCl, ethanol and milli-Q water, and dried overnight at 60 °C under vacuum. The syntheses of redox-active homopolymer, P(4VC)_100_ (*M*_n_ ≈ 9.5 kg/mol and *M*_w_/*M*_n_ ≈ 1.16) and copolymer, P(4VC_86_-*stat*-SS_14_) (*M*_n_ ≈ 13.0 kg/mol and *M*_w_/*M*_n_ ≈ 1.22) bearing catechol pendant was described in our previous publication, and the synthetic scheme is also shown in the supplementary information [30].

Preparation of RAP/CNTs buckypaper electrodes: We fixed the buckypaper electrode composition of RAP:CNTs to 60:40 wt%. We prepared RAP/CNTs buckypaper electrodes of different mass loadings in the range of 0.5 to 30 mg cm^−2^ following our recent articles [30,52]. For instance, buckypaper electrode of 2.5 mg cm^−2^ mass loading is fabricated as following: 28.9 mg of CNTs were dispersed in 20 mL NMP using a tip sonicator, followed by addition of 43.4 mg of RAP, proceeding to sonication for 2 h in a bath sonicator (Branson 2510, 100 W, 42 kHz) and overnight stirring to prepare the electrode ink. The suspension was filtrated through a Nylon filter (pore size ~0.45 μm) with the aid of vacuum, followed by thorough rinsing with NMP to remove loosely bound polymer. The buckypaper was carefully peeled off from the filter and dried overnight at 60 °C under vacuum. The buckypaper was cut into circular discs with a diameter of 12 mm with mass loading of the active-material 2.5 mg cm^−2^.

Preparation of coin cells: Zn||RAP full-cells were assembled using a circular disc (12 mm diameter) of RAP/CNTs as the cathode, Zn foil (0.2 mm thickness, 10 mm diameter) as the anode and a porous Whatman^®^ glass microfiber filters (Grade GF/B) soaked with ≈200 µL of 4 m Zn(TFSI)_2_ as the aqueous electrolyte in CR2032 coin cells. The cells were assembled in a high-purity argon-filled glovebox (MBraun; O_2_ < 1.5 ppm) to avoid any possible contamination by oxygen.

Electrochemical Measurements: The cyclic voltammograms (CVs) of RAPs were obtained using a flooded three-electrode electrochemical cell with RAP/CNTs ink modified glassy carbon (GC, with an area of 0.07 cm^2^), Zn wire and Zn foil as the working, reference and counter electrodes, respectively. The CV of Zn anodic semi-reaction was obtained using a flooded three-electrode electrochemical cell with Ti-foil (with an area of 0.2 cm^2^), Zn wire and Zn foil as the working, reference and counter electrodes, respectively. All the experiments were performed using 4 m Zn(TFSI)_2_ in milli-Q water as aqueous electrolyte. The electrolyte was degassed with argon, and voltammograms were recorded at room temperature under a positive pressure of argon atmosphere. These experiments were carried out with a Bio-logic VMP3 multichannel Potentiostat/Galvanostat (Biologic SP-150). Unless otherwise specified, cycling and rate performance of Zn||RAP batteries in coin-type cells were assessed by galvanostatic charge–discharge (GCD) experiments with a Neware battery cycler at 25 °C. Additionally, these experiments were also realized at variable temperature in the range of 25 to −35 °C using a Binder climatic chamber with a Biologic SP-150. As a commonly used procedure for polymer-based organic batteries, the specific capacities and current rates were normalized with respect to the mass of polymer in the cathode. Electrochemical impedance spectroscopy (EIS) experiments were performed with a Bio-logic VMP3 multichannel potentiostat using coin cells. The EIS data were collected in the 0.01–10^5^ Hz frequency range using a sinusoidal signal with an amplitude of 10 mV (*V*_rms_ ≈ 7.07 mV) at equilibrium discharge potential (1.15 V at 50% depth-of-discharge).

## 3. Results

### 3.1. Design of Zn||Poly(catechol) Aqueous Battery

Figure 1b shows CV of Zn anodic semi-reaction and CVs of P(4VC)_100_ and P(4VC_86_-*stat*-SS_14_) cathode materials using 4 m Zn(TFSI)_2_ as the aqueous electrolyte. As previously demonstrated, a facile and highly reversible Zn plating/stripping process with onset potentials of initial −0.06/+0.01 V (vs. Zn/Zn^2+^) in 4 m Zn(TFSI)_2_ can be observed [51]. Both the homopolymer and copolymers featured well-defined oxidation/reduction peaks at 1.3 and 1.27 V/1.14 and 1.20 V (vs. Zn/Zn^2+^), respectively. As previously demonstrated, these redox processes are associated to the conversion of catecholates to *ortho*-quinones during the oxidation step and reverse reaction happens during the cathodic sweep reducing *ortho*-quinones to catecholates with concomitant Zn^2+^ coordination (See Figure 1a for the simplified redox reaction scheme) [39,40,46,48,51]. Taking advantage of poly(catechol)’s high redox potential, Zn||polymer battery with an anticipated voltage output of ~1.2 V can be potentially constructed by combining Zn anode and poly(catechol) cathode in the aqueous electrolyte (Figure 1b).

### 3.2. Comparative Electrochemical Performance of Zn||P(4VC)_100_ vs. Zn||P(4VC_86_-*stat*-SS_14_) Full-Cells

To evaluate the electrochemical performance of Zn||poly(catechol) full batteries, coin cells were assembled by using poly(catechol) (homopolymer or copolymer) as the composite cathode and Zn foil as anode using 4 m Zn(TFSI)_2_ aqueous electrolyte. It is worth remarking that the composite cathodes are RAP-supported, self-standing, metal current collector- and binder-free CNTs buckypapers (RAP/CNTs ratio = 60/40 (wt/wt%) (see Section 2 for cathode and coin cells preparation).

#### 3.2.1. Comparative Electrochemical Performance at Room Temperature

**Figure 2:**
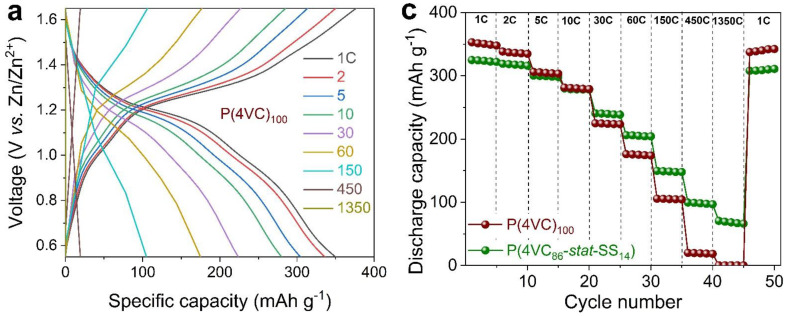

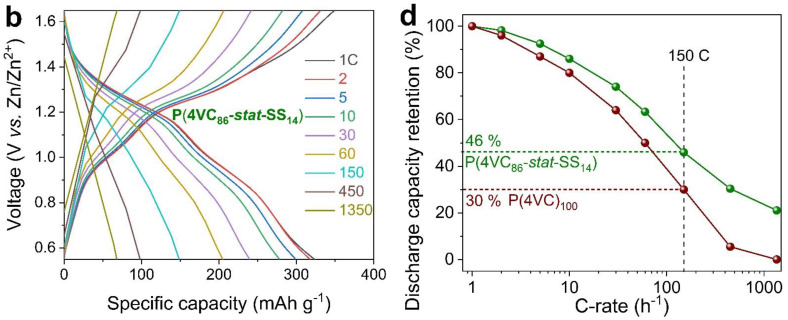
Comparative rate performance of Zn||P(4VC)_100_ and Zn||P(4VC_86_-*stat*-SS_14_) cells at room temperature. (**a**, **b**) Representative specific capacity-voltage profiles of Zn||P(4VC)_100_ (**a**), and Zn||P(4VC_86_-*stat*-SS_14_) (**b**) cells at various C-rates. (**c**) Discharge capacity vs. cycle number, and the corresponding discharge capacity retention at different C-rates (**d**). The discharge capacities at lower C-rates are normalized with respect to the discharge capacity at 1 C.

First, we compared the electrochemical performance of Zn||P(4VC)_100_ and Zn||P(4VC_86_-*stat*-SS_14_) cells with reasonable mass loading electrodes of 2.5 mg cm^−2^ at room temperature. Galvanostatic charge-discharge (GCD) experiments were performed at different C-rates ranging from 1 C (1 h of charge/discharge) to 1350 C (2.67 s of charge/discharge) (Figure 2). It can be observed that at low C-rates (<10 C), the P(4VC_86_-*stat*-SS_14_) copolymer, containing ion-conducting 4-styrenesulfonic acid (SS) units, exhibited lower gravimetric capacity (325–279 mAh g^−1^) than the P(4VC)_100_ homopolymer (350–280 mAh g^−1^) (see Figure 2c). This result was expected since the styrenesulfonic units are non-redox-active groups adding “dead” weight from a capacity perspective and resulting in lower values of theoretical capacity for the copolymer (344 mAh g^−1^ vs. 400 mAh g^−1^ for the homopolymer). Despite the lower capacity values for the copolymer, most notably, capacity utilization was found to be higher than for P(4VC)_100_ in the entire tested current rate (C-rate) range (Figure S1). At increasing C-rates, both the capacities (Figure 2c) and consequently their capacity retentions (capacities at higher C-rates w.r.t. the capacity at 1 C; Figure 2d) decreased monotonically, recovering their initial capacities when cycled again at 1 C. Interestingly, this capacity decrease is much more pronounced in the P(4VC)_100_ homopolymer than in the P(4VC_86_-*stat*-SS_14_) copolymer. For instance, the discharge capacities (and their percent retentions) were 105 (30%) and 149 (46%) mAh g^−1^ at 150 C for P(4VC)_100_ and P(4VC_86_-*stat*-SS_14_), respectively. Interestingly, even at an extreme C-rate of 1350 C (less than 3 s charge/discharge period), Zn||P(4VC_86_-*stat*-SS_14_) still delivered a considerable capacity of 65 mAh g^−1^ (20% capacity retention), where in contrast, Zn||P(4VC)_100_ cell was almost non-functioning. This seems to indicate that the styrenesulfonic units are improving the rate performance of the cathode possibly due to the enhancement of Zn^2+^ ion-conductivity in the bulk electrode. Additionally, when the C-rate was brought back to 1 C, nearly quantitative capacity recovery was observed in both the cases, signifying a strong tolerance of the redox reaction sites for the high currents.

#### 3.2.2. Comparative Electrochemical Performance at Low Temperatures

**Figure 3:**
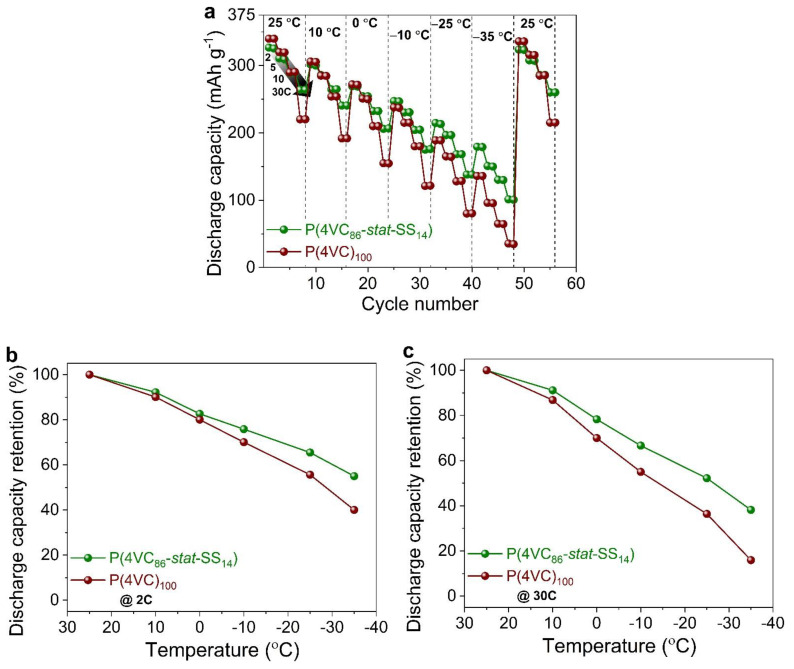
Comparative rate performance of Zn||P(4VC)_100_ and Zn||P(4VC_86_-*stat*-SS_14_) cells at different temperature and at different C-rates. (**a**) Discharge capacity vs. cycle number, recorded at different C-rates (from 2 C to 30 C) and at different temperature (from +25 °C to −35 °C). (**b**,**c**) Discharge capacity retention at 2 C (**b**) and at 30 C (**c**). The discharge capacities at lower temperature are normalized with respect to the discharge capacity at 25 °C.

Then, we extended our comparative electrochemical performance studies to different temperatures, ranging from +25 to −35 °C (Figure 3; Supplementary Figures S2 and S3). Once again, Zn||P(4VC_86_-*stat*-SS_14_) demonstrated superior rate performance not only at higher C-rates (for a given temperature), but also at lower temperatures (for a given C-rate) compared to Zn||P(4VC)_100_. For example, as shown in Figure 3b,c, capacity retention (compared to the capacity at +25 °C) at lower temperatures was higher for Zn||P(4VC_86_-*stat*-SS_14_) than for Zn||P(4VC)_100_ at both 2 C and 30 C. Remarkably, at rather low temperatures of −25 and –35 °C, and at a high C-rate of 30 C, Zn||P(4VC_86_-*stat*-SS_14_) yet attained high discharge capacities of 138 and 101 mAh g^−1^, respectively, that correspond to 52 and 38% capacity retention compared to the capacity at +25 °C versus low capacities of 80 and 35 mAh g^−1^ (36 and 16% capacity retention), respectively, compared to the Zn||P(4VC)_100_. Furthermore, after low-temperature experiments, when the operational temperature was reset back to +25 °C, the capacities for both cells were almost recovered to their original values, suggesting negligible intolerance of cell components towards such low temperatures.

### 3.3. Electrochemical Kinetic Evaluation of Redox Reactions

**Figure 4:**
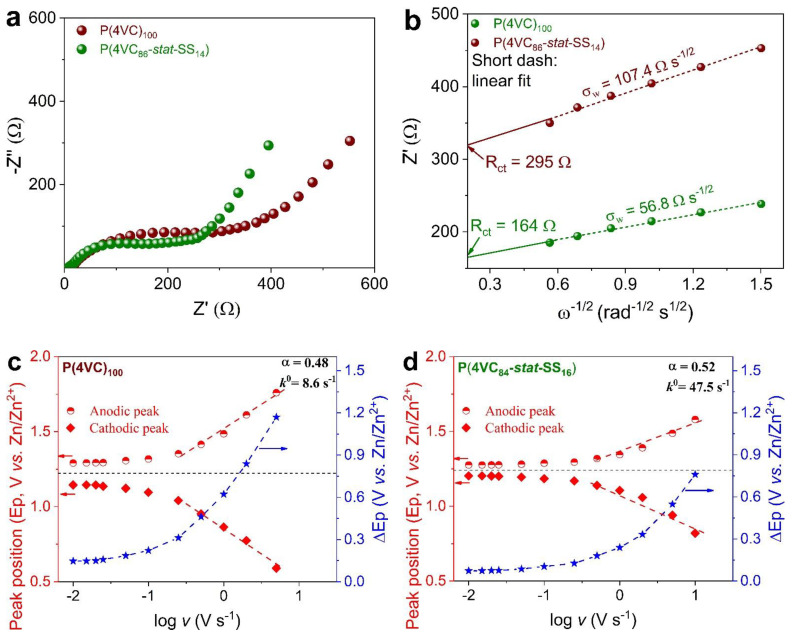

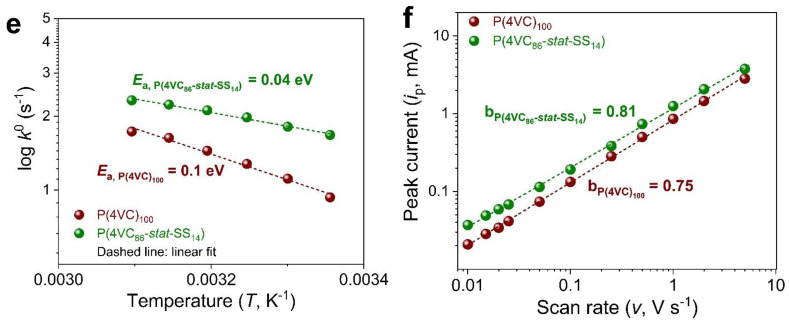
Comparative electrochemical kinetic analyses of redox reaction between P(4VC)_100_ and P(4VC_86_-*stat*-SS_14_). (**a**) Nyquist plots, and (**b**) plots showing the correlation between real (Z′)-part of complex impedance and *ω*^−1/2^ in the semi-infinite diffusion region to calculate the apparent diffusion coefficient in full-cells. (**c**,**d**) Variation of anodic and cathodic peak positions (Ep), and peak separation (ΔEp) as a function of the scan rate (in logarithmic scale) to calculate rate constant. (**e**) A fitting of temperature dependence of rate constant with the Arrhenius equation to calculate activation energy. (**f**) Peak current vs. scan rate in logarithmic scale for the cathodic peak to obtain b-values according to *i*_p_ = a*v*^b^.

In order to elucidate the observed rate performance differences between both the redox polymers, CV and EIS analyses were conducted on the Zn||poly(catechols) coin cells. Figure 4a compares the Nyquist plots of the buckypaper electrodes that feature two well-defined regions: a depressed semicircle in the high frequency region and an inclined line in the low-frequency that are connected by Warburg impedance (σ_w_), represented by a line of ~45° slope. A nearly vertical tail in the low-frequency region is associated with the diffusion of Zn^2+^ ions in the bulk of the electrode. 

Charge-transfer resistance (R_ct_) obtained as the extrapolation of Z’ versus ω^−1/2^ linear regression plot (Figure 4b) according to the Equation (1) was found to be lower for P(4VC_86_-*stat*-SS_14_) (164 Ω) than the P(4VC)_100_ (295 Ω).

|Z’| = R_s_ + R_ct_ + σ_w_ω^−1/2^ (1)

where Z’ is the real-part of complex impedance, R_s_ is the equivalent series resistance, R_ct_ is the charge transfer resistance, and ω is the angular frequency (ω = 2πf).

Furthermore, apparent Zn^2+^ ion diffusion coefficient (*Dapp*), calculated according to Equation (2) was higher for P(4VC_86_-*stat*-SS_14_) (1.37 × 10^−8^ cm^2^ s^−1^) than its homopolymer analogue (0.38 × 10^−8^ cm^2^ s^−1^).

(2) $$Dapp=\frac{R^{2}T^{2}}{2A^{2}n^{4}F^{4}C^{2}\sigma_{w}{}^{2}}$$

where *R* is the molar gas constant, *T* is the absolute temperature, *A* is the surface area of the electrode material, *n* is the number of electrons involved in the electrode reaction, *F* is the Faraday constant, *C* is the Zn^2+^ concentration within the working electrode and σ_w_ is the coefficient of the Warburg element, which is related to the real-part of the impedance according to the Equation (1).

The CV analysis of both polymers over a wide range of scan rates from 0.01 to 100 V s^−1^ was carried out to determine other kinetic parameters of redox reactions (see Figure S4 for CVs). The P(4VC_86_-*stat*-SS_14_) was characterized by a smaller peak-to-peak voltage separation for all the given scan rates than the P(4VC)_100_, indicating faster kinetics for the copolymer. Quantitatively, apparent reaction rate constant (*k*^0^), determined from the scan rate-dependence of peak potential using the Laviron method [53] was found to be about 5.5-fold higher for P(4VC_86_-*stat*-SS_14_) (47.5 s^−1^) than the P(4VC)_100_ (8.6 s^−1^) (Figure 4c,d). Furthermore, Laviron approach was extended to study the dependence of formal potentials and thus *k*^0^ on the temperature (from 25 to 50 °C). An energy barrier activation energy (*E*_a_) parameter, calculated using the negative slope of Arrhenius plots (Figure 4e, Equation (3)) was lower for P(4VC_86_-*stat*-SS_14_) (0.04 eV) than the P(4VC)_100_ (0.1 eV) [54].

*k*^0^ = Ae^−*E*^_a_^/(*RT*)^ (3)

Finally, the peak currents (*i*_p_) in the CV curves as a function of the scan rate (*v*) obeys a power-law relationship as: *i*_p_ = a*v*^b^; where, a and b are adjustable coefficients [55]. The exponential b-value can be determined by the slope of log (*i*_p_) vs. log (*v*) plot for the redox processes. Ideally, the b-value of 0.5 indicates a diffusion-controlled process, whereas the b-value of 1.0 is the signature of a capacitive-controlled behaviour. Here, the capacitive-type electrochemical response is assumed to have mainly originated from bulk electrochemical reaction sites (similar to intercalation pseudocapacitance that is observed in some inorganic intercalation compounds, which is different from conventional surface pseudocapacitance in nanomaterials) that are less limited by the diffusion processes on account of polymer’s high reaction rates and superior ion mobilities [30,55,56]. The b-values of 0.75 and 0.81 were obtained for P(4VC)_100_ and P(4VC_86_-*stat*-SS_14_), respectively (Figure 4f). b-values in the range of 0.75–0.81 for both polymers indicate that a mixed electrochemical reaction kinetics is operative, but the overall response tends to be less diffusion-limited redox processes in the case of P(4VC_86_-*stat*-SS_14_) (higher b-value of 0.81). On the contrary, a lower b-value of 0.75 suggests that the redox processes are more diffusion-controlled for homopolymer P(4VC)_100_.

### 3.4. Development of More Practical Batteries Using High Mass Loading Electrodes 

Motivated by the improved superior electrochemical performance of Zn||P(4VC_86_-*stat*-SS_14_) over the Zn||P(4VC)_100_ at 2.5 mg cm^−2^ polymer mass loading, we were encouraged to prepare even higher mass loading electrodes, targeting at further boost the capacity values at the electrode level. Once again, we adapted buckypaper approach to construct different mass loading poly(catechol) electrodes from 0.5 to 30 mg cm^−2^ (see Supplementary Figure S5 for representative digital images of different mass loading copolymer electrodes). 

**Figure 5:**
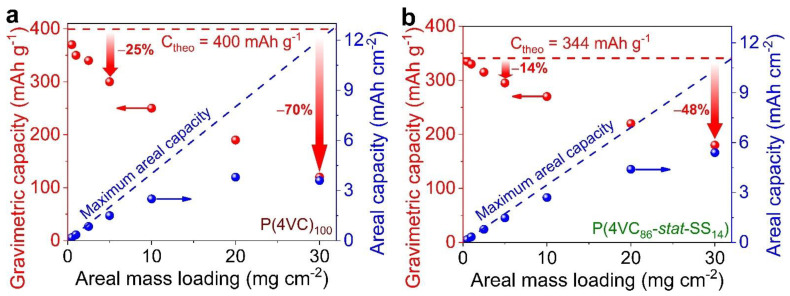
Comparative electrochemical performance of Zn||P(4VC)_100_ and Zn||P(4VC_86_-*stat*-SS_14_) cells at different mass loadings. (**a**,**b**) Gravimetric/areal capacities of P(4VC)_100_ (**a**) and P(4VC_86_-*stat*-SS_14_) (**b**) as a function of mass loading.

When we compared the gravimetric capacity of these full-cells at various mass loadings, it was found that Zn||P(4VC_86_-*stat*-SS_14_) exhibited lower gravimetric capacity values (295–335 mAh g^−1^ vs. 300–370 mAh g^−1^) than the Zn||P(4VC)_100_, below 5 mg cm^−2^ (Figure 5a,b). Interestingly, mass utilization for Zn||P(4VC_86_-*stat*-SS_14_) was higher than the Zn||P(4VC)_100_ in the entire mass loading range (see Supplementary Figure S6a,c, for voltage profiles), particularly beyond 5 mg cm^−2^. For instance, at 30 mg cm^−2^, the Zn||P(4VC_86_-*stat*-SS_14_) demonstrated an enhanced mass utilization of 52% (180 mAh g^−1^) versus only a 30% (120 mAh g^−1^) for Zn||P(4VC)_100_.

**Figure 6:**
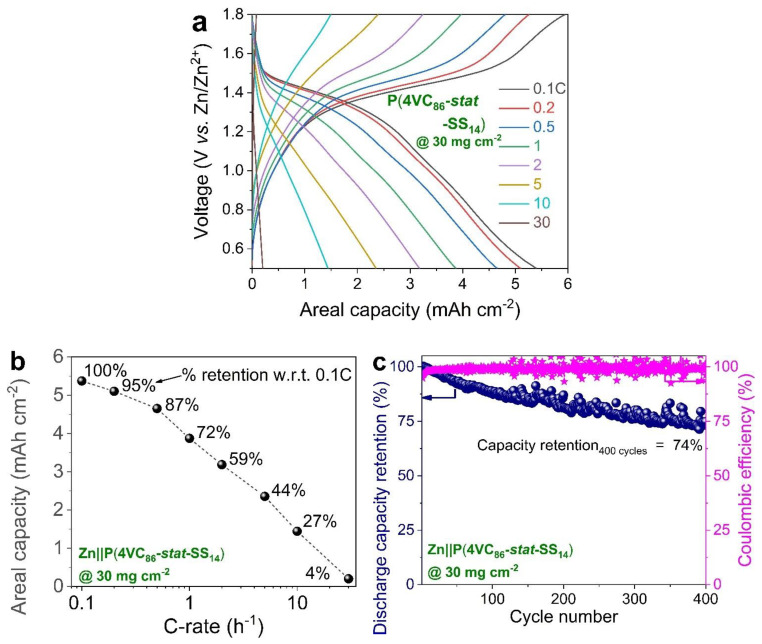
Electrochemical performance of Zn||P(4VC_86_-*stat*-SS_14_) cell at a high mass loading of 30 mg cm^−2^. (**a**,**b**) rate performance: representative specific capacity-voltage profiles (**a**), and the corresponding areal capacity retention at different C-rates (**b**). The discharge capacities at higher C-rates are normalized with respect to the discharge capacity at 0.1 C. (**c**) Cyclic performance: discharge capacities and Coulombic efficiencies measured at 1 C.

Moreover, when the capacities were normalized by per unit area of the electrode, areal capacity (mAh cm^−2^) for Zn||P(4VC_86_-*stat*-SS_14_) scaled almost linearly with the mass loading below 10 mg cm^−2^ due to its superior mass utilization, while significant deviation was observed in the case of P(4VC)_100_, particularly at higher mass loadings (Figure 5a,b). Promisingly, Zn||P(4VC_86_-*stat*-SS_14_) was able to attain the highest areal capacity of 5.4 versus 3.6 mAh cm^−2^ for Zn||P(4VC)_100_ at 30 mg cm^−2^. despite the high mass loading (i.e., 30 mg cm^−2^), Zn||P(4VC_86_-*stat*-SS_14_) was able to deliver satisfactory rate performance, attaining a high areal capacity of 1.5 mAh cm^−2^ at a C-rate as high as 10 C (~3.5 A g^−1^ or ~102 mA cm^−2^) (Figure 6a), that corresponds to 27% capacity retention compared to the capacity at 0.1 C (Figure 6b). This full-cell was also cycled reasonably, retaining 74% of its initial capacity over 400 cycles at 1 C (Figure 6c and Supplementary Figure S7). It is also worth mentioning here that, after the initial few activation cycles, the average Coulombic in the cycling tests remained above 99%.

## 4. Discussion

In our previous studies, we demonstrated that by incorporating cation conducting anionic comonomer pendants (e.g., sulfonates) within the polymer chain, the electrochemical performance was drastically improved compared to its homopolymer analogue [30,37] in electrolytes containing monovalent charge carriers (H^+^, Li^+^, etc.). In this study, we extend the investigation also to multivalent cation-based electrolytes, specifically we affirmed that this trend is also valid for Zn^2+^ cation as the charge carriers in a Zn-metal battery.

First, the comparative rate capability studies between Zn||P(4VC)_100_ and Zn||P(4VC_86_-*stat*-SS_14_) full-cells revealed superior dynamic performance in the case of copolymer not only at the room temperature, but also at lower temperatures (up to −35 °C). Thanks to the low meting temperature of 4 m Zn(TFSI)_2_ (i.e., −38 °C), we successfully achieved the low-temperature operativity for both the polymers even way below the freezing point of water [51]. Note that most of the conventional low-to-moderately concentrated zinc based aqueous electrolytes generally will not offer such a low melting temperature window.

Second, a series of electrochemical kinetic parameters were evaluated by CV and EIS in order to interpretate the observed rate performance differences. Sulfonate comonomer-mediated smaller energy barrier (lower *E*_a_) for the redox reactions, lower charge-transfer resistance and facilitated Zn^2+^ ion mobility (higher *Dapp*). All these parameters are thought to be reflected in the higher calculated rate constant for the P(4VC_86_-*stat*-SS_14_). Consequently, Zn||P(4VC_86_-*stat*-SS_14_) delivered boosted rate performance on account of higher non-diffusion-controlled current responses than the Zn||P(4VC)_100_. The coordination and/or conduction of Zn^2+^ cations with the sulfonate anionic groups in the copolymer electrode layer is assumed to play this crucial role to enhance the electrochemistry, and thus the performance. Very recently, Lee et al. [57] and Zhi et al. [58] demonstrated a similar electrochemical performance enhancement strategy but applied to Zn^2+^ conducting electrolytes based on a zinc sulfonated covalent organic framework and zwitterionic sulfobetaine hydrogels, respectively.

**Figure 7:**
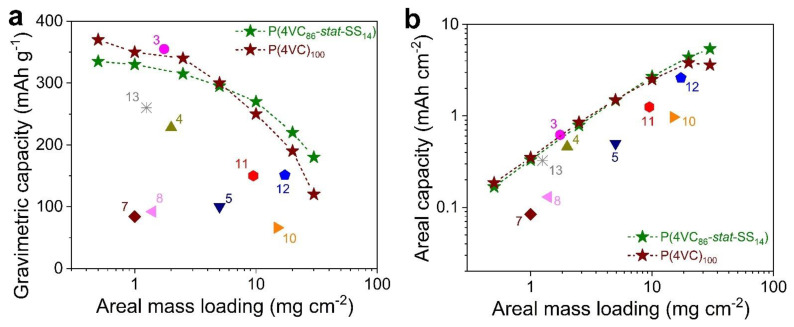
Comparing gravimetric (**a**) and areal capacity (**b**) of Zn||P(4VC)_100_ and Zn||P(4VC_86_-*stat*-SS_14_) cells with the state-of-the-art polymer electrodes in AZPBs (Table S1).

Third, we compared both the gravimetric and areal capacities of these polymers with the state-of-the-art AZPBs comprising polymeric cathodes at different mass loadings (Figure 7a,b and Supplementary Table S1). The capacities of our polymers are superior to most of the reported systems. The aforementioned electrochemical enhancement for the copolymer should also hold a great prospect to prepare high mass loading electrodes. Most often, electron and/or ion diffusion limitations within the bulk of thick electrodes limit capacity utilization of the active material, especially at high current rates, which was actually not the case with our copolymer in comparison with the homopolymer. Optimistically, our copolymerization strategy enabled us to push the mass loading to a high value (30 mg cm^−2^), and attained competitive areal capacity of 5.4 mAh cm^−2^, which is the highest value reported till date for organic cathodes in AZPBs (Figure 7b). This high value of areal capacity is particularly encouraging from the perspective reducing the cost of a practical battery by simultaneously minimizing the inactive components in the battery and maximizing the capacity at the cell level [52].

## 5. Conclusions

In this article, we successfully demonstrated that the macromolecular engineering is a powerful tool to precisely tune and optimize the electrochemical properties and performances of the polymeric active-materials. Incorporation of Zn^2+^ ion coordinating and/or conducting sulfonate anionic groups in the copolymer electrode of P(4VC_86_-*stat*-SS_14_) drastically improved the Zn||polymer cell performance, in terms of superior capacity utilization (and thus, higher specific capacity) at low temperatures (up to −35 °C) and at high mass loading electrodes (up to 30 mg cm^−2^) compared to the homopolymer, P(4VC)_100_. The latter enhancement is particularly encouraging which enabled us to boost the areal capacity for the copolymer to a high value of 5.4 vs. 3.6 mAh cm^−2^ for P(4VC)_100_. We hypothesize that our approach not only paves ways toward the design of a “high-performance” and “advanced” organic cathode, but also may presumably leads to the development of safe, environmentally benign, and practical organic batteries by taking advantage of the abundant Zn metal anode and the high safety of aqueous electrolyte. We also believe that the presented strategy may inspire polymer chemists to translate the macromolecular engineering knowledge that is hardly applied in a limited number of works to design innovative organic electrodes with superior electrochemical properties.

## Data Availability

Data is contained within the article or Supplementary Material.

## Supplementary Materials

The following are available online at https://www.mdpi.com/article/10.3390/polym13111673/s1, Table S1. An exhaustive list comprising the comparison of Zn||P(4VC)_100_ and Zn||P(4VC_86_-*stat*-SS_14_) cell performances with the *stat*e-of-the-art Zn||polymer aqueous batteries. Figure S1. Capacity utilization of Zn||P(4VC)_100_ and Zn||P(4VC_86_-*stat*-SS_14_) cells at various C rates. The capacity utilization values were obtained dividing (practical capacity * 100) by theoretical capacity. Theoretical specific capacity of P(4VC)_100_ and P(4VC_86_-*stat*-SS_14_) are 400 and 344 mAh g^−1^, respectively. Figure S2. Representative specific capacity–voltage profiles of Zn||P(4VC)_100_ cells at various C-rates (from 2 C to 30 C), recorded at +25 (a), +10 (b), 0 (c), −10 (d), −25 (e), and −35 °C (f). Figure S3. Representative specific capacity–voltage profiles of Zn||P(4VC_86_-*stat*-SS_14_) cells at various C-rates (from 2 C to 30 C), recorded at +25 (a), +10 (b), 0 (c), −10 (d), −25 (e), and −35 °C (f). Figure S4. Cyclic voltammograms of P(4VC)_100_ (a) and P(4VC_86_-*stat*-SS_14_) (b) composite cathodes at different scan rates. The CVs are normalized by the peak anodic current to show the peak shift with the scan rates. WE = RAP/CNTs deposited on glassy carbon, RE = Zn wire, and CE = Zn foil. Figure S5. Representative digital images of copolymer buckypaper electrodes of different polymer mass loadings, along with their thickness. Figure S6. Comparing gravimetric capacity, along with mass utilization (based on discharge capacity) (a, c) and areal capacity (b, d) of Zn||P(4VC)_100_ (a, b) and Zn||P(4VC_86_-*stat*-SS_14_) (c, d) cells with different mass loadings. Figure S7. Cyclic performance of Zn||P(4VC_86_-*stat*-SS_14_) at a high mass loading of 30 mg cm^−2^, showing the rrepresentative normalized capacity–voltage profiles over 400 GCD cycles at 1 C. References [38,39,40,41,42,43,44,45,46,47,48,49,50] are cited in the supplementary materials.
