# Supplementary material for: Macromolecular Engineering of Poly(catechol) Cathodes towards High-Performance Aqueous Zinc-Polymer Batteries
Source: Polymers (Basel). 2021 May 21;13(11):1673. doi: 10.3390/polym13111673 (PMC8196623; doi:10.3390/polym13111673)
Supplement: Supplementary file 1 [file polymers-13-01673-s001.zip › polymers-1231039-SI.pdf]

polymers MDPI

Supplementary Information

Macromolecular Engineering of Poly(catechol) Cathodes towards High-Performance Aqueous Zinc-Polymer Batteries

Nagaraj Patil *, Jesus Palma and Rebeca Marcilla *

Electrochemical Processes Unit, IMDEA Energy Institute, Avda. Ramón de la Sagra 3, 28935 Móstoles, Spain; jesus.palma@imdea.org

* Correspondence: nagaraj.patil@imdea.org (N.P.); rebeca.marcilla@imdea.org (R.M.); Tel.: +34-917371131 (R. M.)

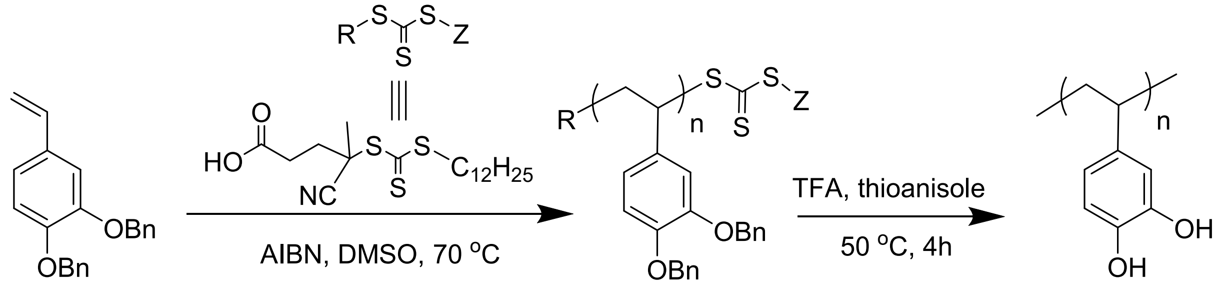

4VCⓟ P(4VCⓟ) P(4VC)

Scheme S1. Synthetic scheme of poly(4-vinyl catechol) homopolymer ($P(4VC)_{100}$) by RAFT polymerization.

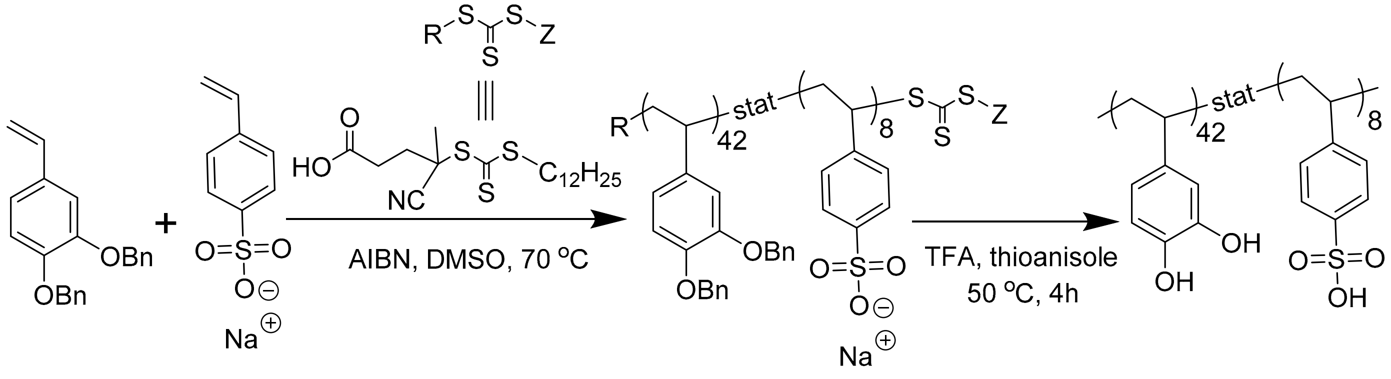

4VCⓟ SSNa $P(4VCⓟ_{42}$-***stat***-$SSNa_8)$ $P(4VC_{42}$-***stat***-$SS_8)$

Scheme S2. Synthetic scheme of poly(4-vinyl catechol-*stat*-4-styrenesulfonic acid sodium salt) copolymer ($P(4VC_{86}$-*stat*-$SS_{14})$) by RAFT polymerization.

Table S1. An exhaustive list comprising the comparison of Zn||P(4VC)$_{100}$ and Zn||P(4VC$_{86}$-*stat*-SS$_{14}$) cell performances with the state-of-the-art Zn||polymer aqueous batteries.

Active-material	#[ref]	$C_{s,\,theo}$; $C_{s,\,initial/max.}$; capacity utilization ratio (%)	cycle performance: % $C_{s,\,retention}$; number of cycles; speed (C-rate or current density)	average discharge potential (V, vs Zn/Zn^{2+})	cell configuration: anode | electrolyte | cathode (active-material/conducting additive/binder, wt/wt/wt%)
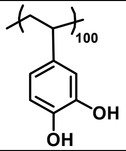	this work	400<br>350<br>87.5%	n a	1.2	Zn | 4 m Zn(TFSI)$_2$ | P(4VC)$_{100}$<br>(P(4VC)$_{100}$ / MWCNTs, 60/40)
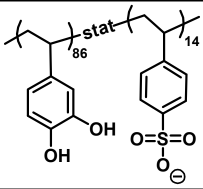	this work	344<br>324<br>94%	74%<br>400<br>1C	1.2	Zn | 4 m Zn(TFSI)$_2$ | P(4VC$_{86}$-*stat*-SS$_{14}$)<br>(P(4VC$_{86}$-*stat*-SS$_{14}$) / MWCNTs, 60/40)
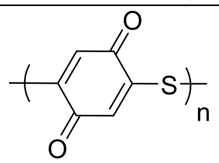	1[38]	388<br>203<br>52%	86%<br>50<br>0.2C	1	Zn | 3M Zn(OTf)$_2$ | 1<br>(1/carbon/PVDF, 60/30/10)
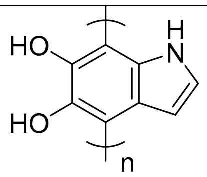	2[39]	n a<br>126<br>n a	96%<br>500<br>0.2 A/g	0.9	Zn | 3.3M ZnSO$_4$ | 2<br>(2/MWCNT, 38/62)
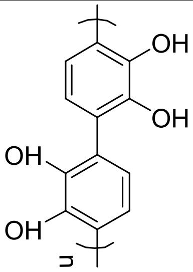	3[40]	500<br>355<br>71%	74.4%<br>3000<br>2C	0.8	Zn | 3M Zn(OTf)$_2$ | 3+graphene<br>(3+graphene/AB/PVDF, 70/20/10)
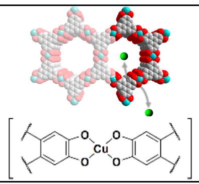	4[41]	250<br>228<br>91%	75%<br>500<br>4A/g	0.75	Zn | 3M Zn(OTf)$_2$ | 4<br>(4/AB/PVDF, 60/20/20)
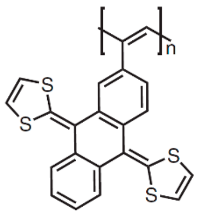	5[42]	133<br>100<br>75%	86%<br>10000<br>10C	1.1	Zn | 1M Zn(BF$_4$)$_2$ | 5<br>(5/MWCNT, 50/50)

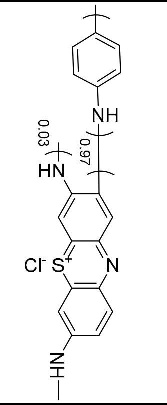	6[43]	n a<br>306*<br>n a<br>* = protons contribute to majority of charge storage	73%<br>1100<br>4 mA/cm^2	1.15<br>sloping	Zn nanoparticles | 2M $ZnCl_2$ + 3M NH_4Cl | 6<br>(electrodeposited 6)
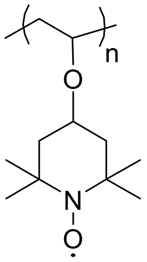	7[44]	117<br>84<br>72%	77%<br>1000<br>1A/g	1.58	Zn | 1M $Zn(OTf)_2$ | 7<br>(7/super P/PVDF, 50/40/10)
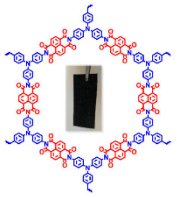	8[45]	n a<br>91<br>n a	n a	0.4	Zn | 2 M $ZnSO_4$ | 8<br>(n a)
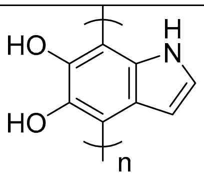	9[46]	n a<br>372<br>n a	60%<br>3500<br>1 A/g	Sloping<br>(0.9)	Zn | 3 M $Zn(OTf)_2$ PVA| 9<br>(n a)
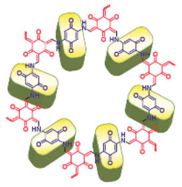	10[47]	442<br>276<br>62%	87%<br>1000<br>3.75 A/g	0.87	Zn | 3 M $ZnSO_4$ | 10<br>(10/CNF/nafion, 76/19/5)
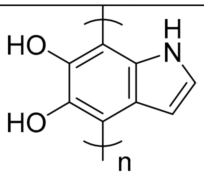	11[48]	n a<br>150<br>n a	100%<br>10000<br>10 mA/cm^2	0.8	Zn | 2 M $ZnSO_4$ | 11<br>(n a)
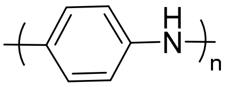	12[49]	n a<br>151<br>n a	100%<br>2000<br>0.2 A/g	0.91	Zn | 7.5 m $ZnCl_2$ | 12<br>(12/KB/PTFE, 60/35/5)
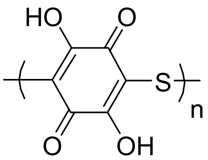	13[50]	308<br>260<br>84%	79%<br>2000<br>2 A/g	0.7	Zn | 2 M $ZnSO_4$ | 13<br>(13/AB/PVDF, 80/10/10)

n a = not available. Theoretical capacity ($C_{s,\,theo}$) = (26801 x n) / MW; n = number of electrons involved in the redox process(es), MW = molecular weight of the active-material. $C_{s,\,initial/last/max.}$ = initial / last cycle / maximum specific discharge capacity obtained during cycling performance. All the specific capacities are expressed in mAh g^{-1}. Material activity = ($C_{s,\,max.}$ / $C_{s,\,theo}$) x 100. Capacity retention (%) = ($C_{s,\,max.\ or\ first\ cycle}$ / $C_{s,\,last\ cycle}$) x 100. nC-rate designates that the current chosen will charge/discharge the battery in 1/n hour.

Zn-salts: zinc trifluoromethanesulfonate ($Zn(OTf)_2$), zinc bis(trifluoromethanesulfonyl)imide ($Zn(TFSI)_2$), zinc sulfate ($ZnSO_4$), zinc tetrafluoroborate ($Zn(BF_4)_2$), zinc chloride ($ZnCl_2$).

Supplementary salts: ammonium chloride (NH_4Cl).

Conducting additives: Ketjen black (KB), carbon black (CB), acetylene black (AB), multi-walled carbon nanotubes (MWCNTs).

Binders: poly(vinylidenedifluoride) (PVDF), poly(tetrafluoroethylene) (PTFE).

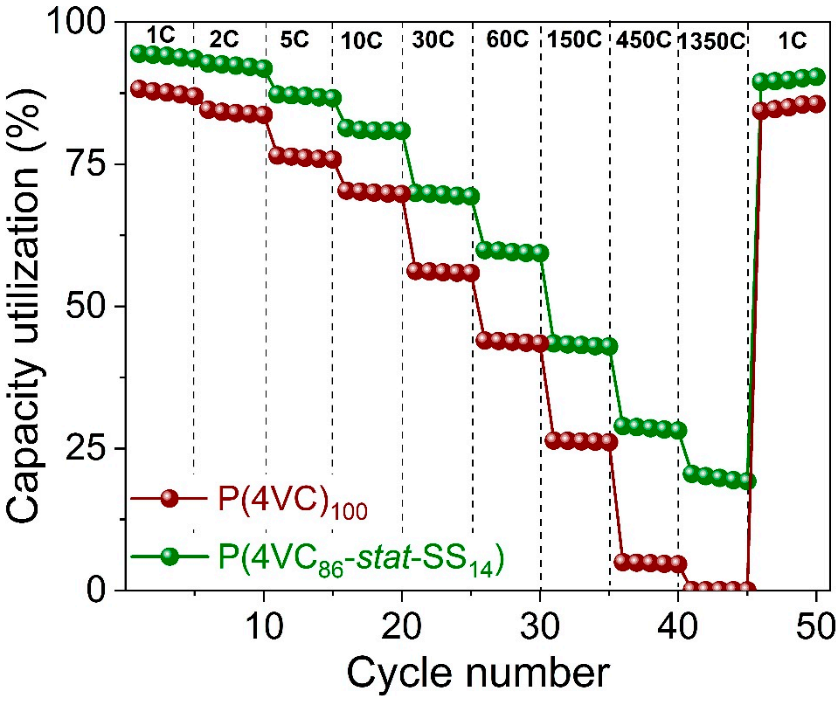

Figure S1. Capacity utilization of Zn||P(4VC)$_{100}$ and Zn||P(4VC$_{86}$-*stat*-SS$_{14}$) cells at various C rates. The capacity utilization values were obtained dividing (practical capacity * 100) by theoretical capacity. Theoretical specific capacity of P(4VC)$_{100}$ and P(4VC$_{86}$-*stat*-SS$_{14}$) are 400 and 344 mAh g^{-1}, respectively.

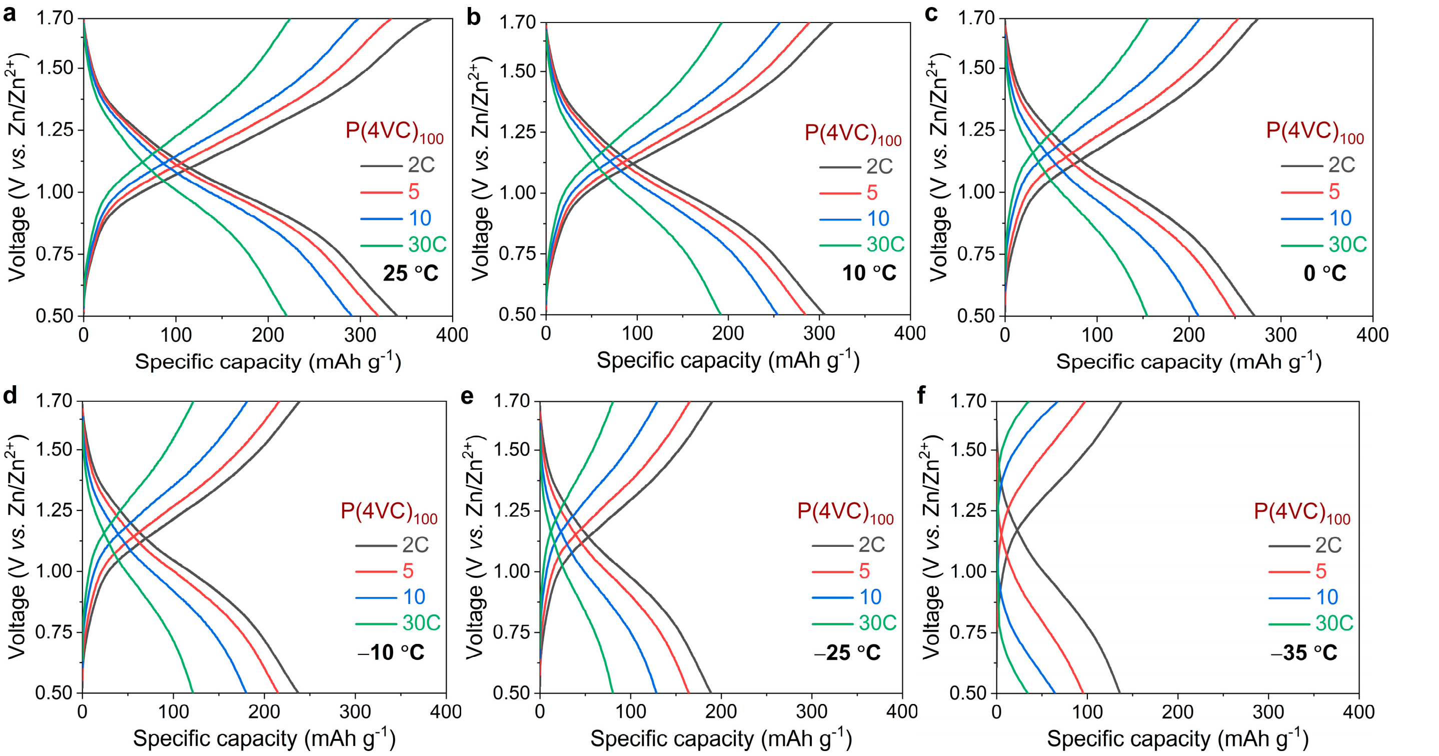

Figure S2. Representative specific capacity–voltage profiles of Zn||P(4VC)$_{100}$ cells at various C-rates (from 2C to 30C), recorded at +25 (a), +10 (b), 0 (c), −10 (d), −25 (e), and −35 °C (f).

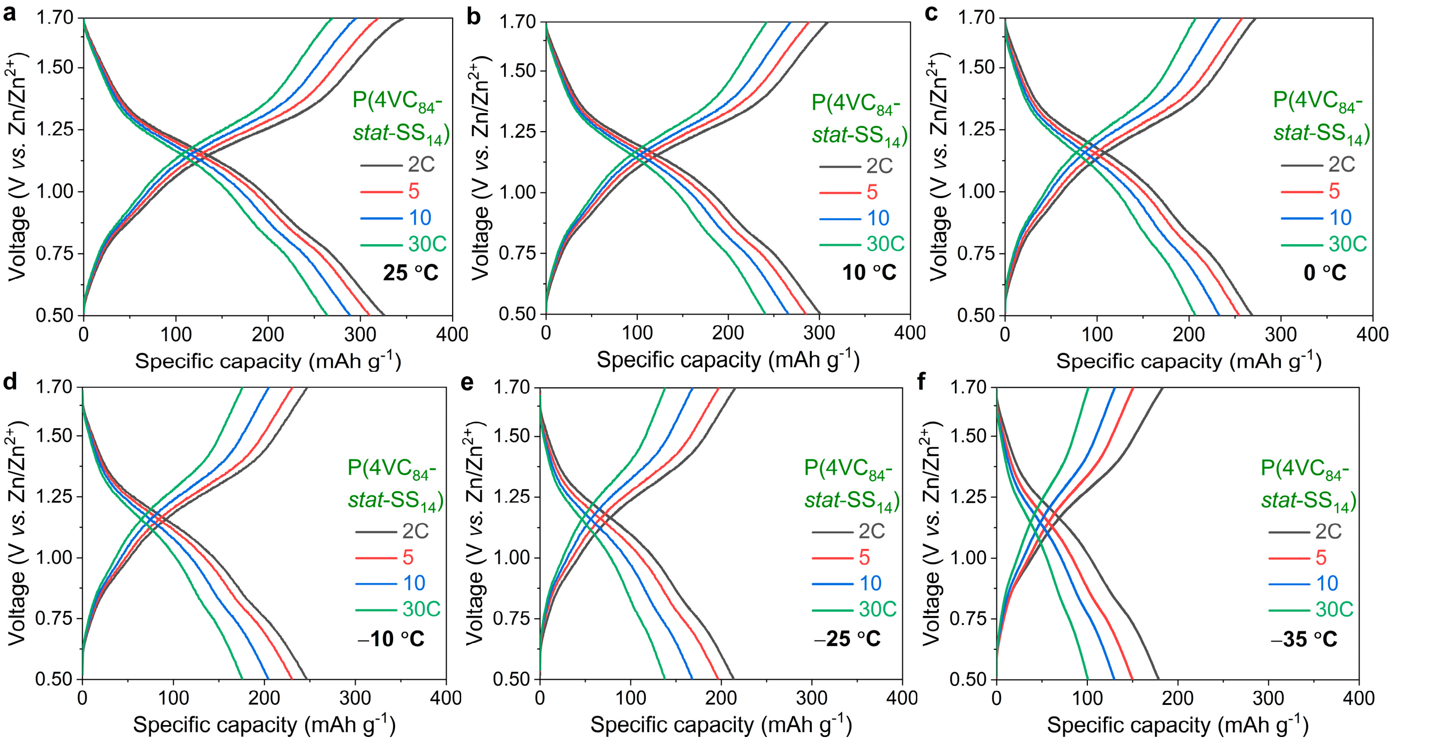

Figure S3. Representative specific capacity–voltage profiles of Zn||P(4VC$_{86}$-*stat*-SS$_{14}$) cells at various C-rates (from 2C to 30C), recorded at +25 (a), +10 (b), 0 (c), −10 (d), −25 (e), and −35 °C (f).

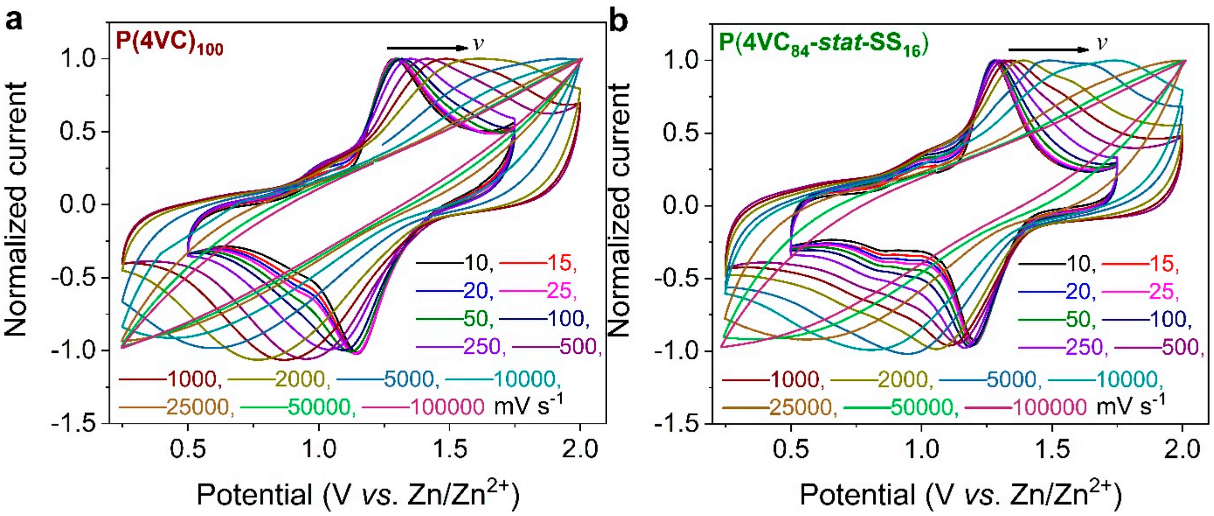

Figure S4. Cyclic voltammograms of $P(4VC)_{100}$ (a) and $P(4VC_{86}$-*stat*-$SS_{14})$ (b) composite cathodes at different scan rates. The CVs are normalized by the peak anodic current to show the peak shift with the scan rates. WE = RAP/CNTs deposited on glassy carbon, RE = Zn wire, and CE = Zn foil.

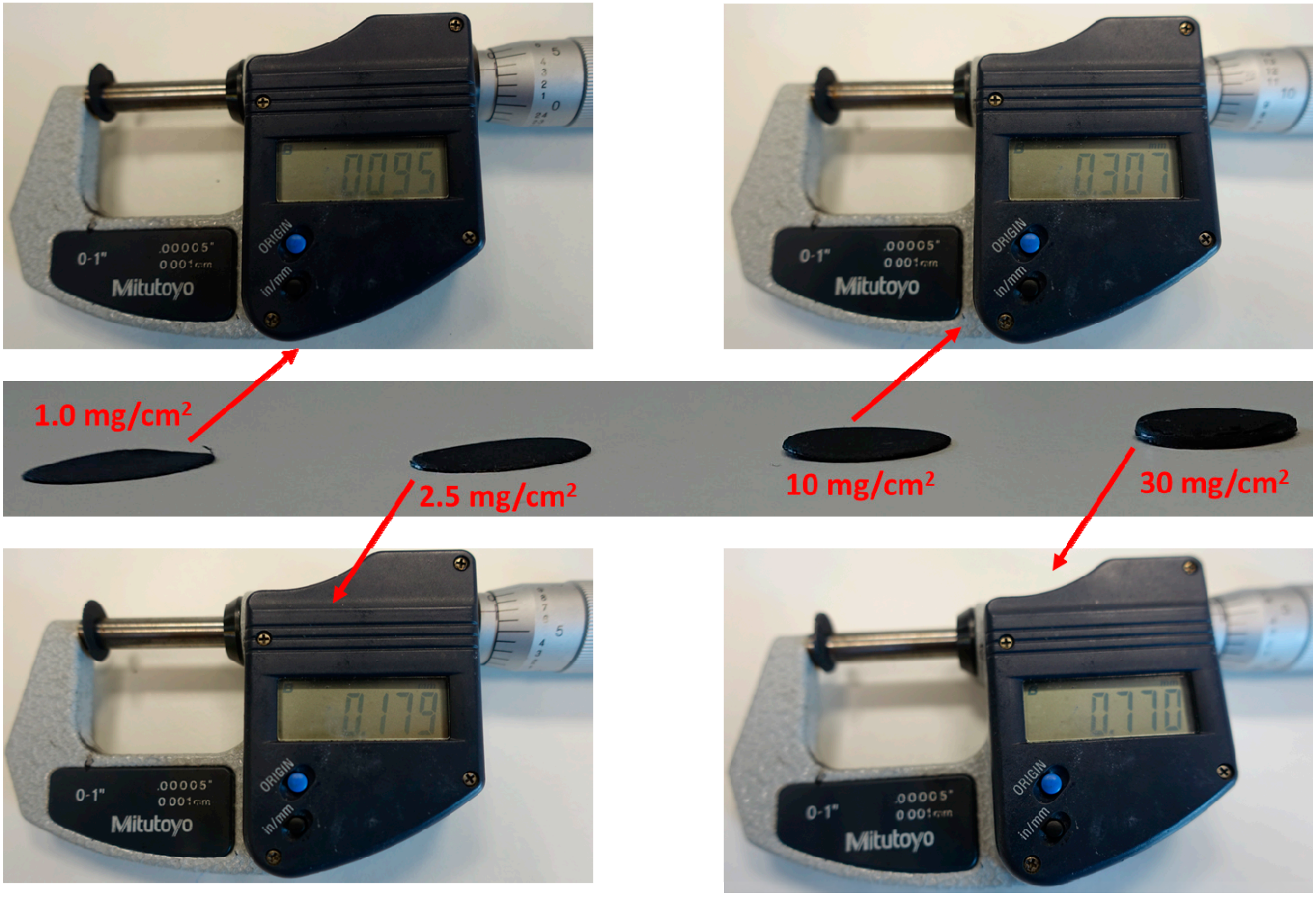

Figure S5. Representative digital images of copolymer buckypaper electrodes of different polymer mass loadings, along with their thickness.

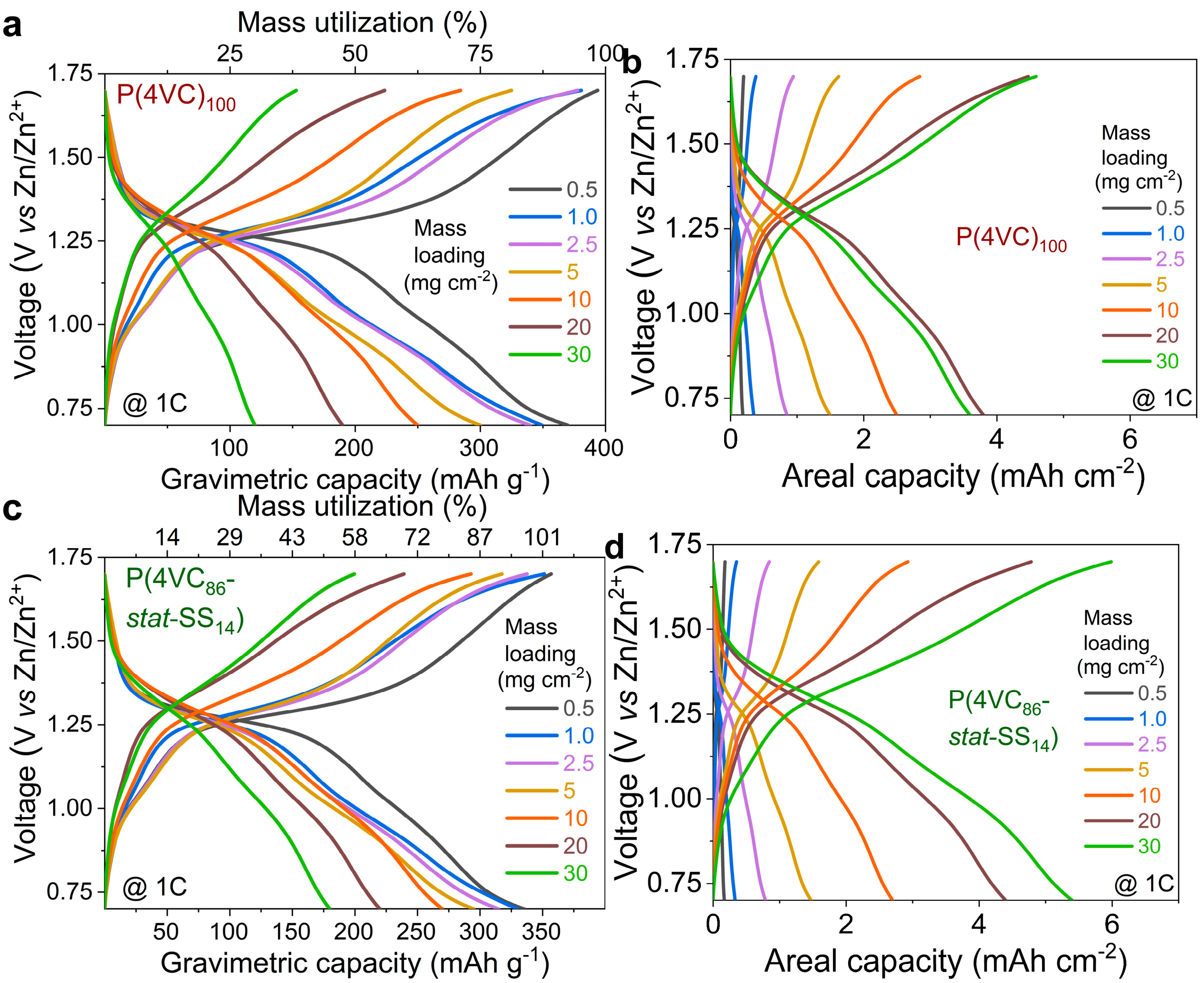

Figure S6. Comparing gravimetric capacity, along with mass utilization (based on discharge capacity) (a, c) and areal capacity (b, d) of Zn||P(4VC)$_{100}$ (a, b) and Zn||P(4VC$_{86}$-*stat*-SS$_{14}$) (c, d) cells with different mass loadings.

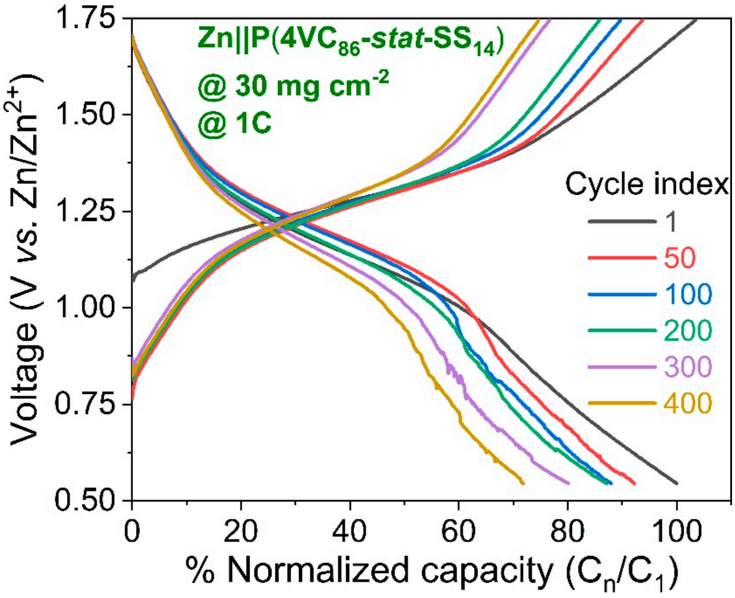

Figure S7. Cyclic performance of Zn||P(4VC$_{86}$-*stat*-SS$_{14}$) at a high mass loading of 30 mg cm^{-2}, showing the representative normalized capacity–voltage profiles over 400 GCD cycles at 1C.
